# β1 integrin is a sensor of blood flow direction

**DOI:** 10.1242/jcs.229542

**Published:** 2019-06-03

**Authors:** Ioannis Xanthis, Celine Souilhol, Jovana Serbanovic-Canic, Hannah Roddie, Antreas C. Kalli, Maria Fragiadaki, Raymond Wong, Dhruv R. Shah, Janet A. Askari, Lindsay Canham, Nasreen Akhtar, Shuang Feng, Victoria Ridger, Jonathan Waltho, Emmanuel Pinteaux, Martin J. Humphries, Matthew T. Bryan, Paul C. Evans

**Affiliations:** 1 Department of Infection, Immunity and Cardiovascular Disease, INSIGNEO Institute for In Silico Medicine, and the Bateson Centre, University of Sheffield, Sheffield S10 2TN, UK; 2 Leeds Institute of Medical Research at St James's and Astbury Centre for Structural Molecular Biology, University of Leeds, Leeds LS2 9JT, UK; 3 Faculty of Biology, Medicine & Health, University of Manchester, Manchester M13 9PL, UK; 4 Wellcome Trust Centre for Cell-Matrix Research, Faculty of Biology, Medicine & Health, University of Manchester, Manchester M13 9PL, UK; 5 Department of Oncology and Metabolism, University of Sheffield, Sheffield S10 2TN, UK; 6 Department of Molecular Biology and Biotechnology, University of Sheffield, Sheffield S10 2TN, UK

**Keywords:** Shear stress, β1 integrin, Blood flow, Endothelial cell, Atherosclerosis, Mechanoreceptor

## Abstract

Endothelial cell (EC) sensing of fluid shear stress direction is a critical determinant of vascular health and disease. Unidirectional flow induces EC alignment and vascular homeostasis, whereas bidirectional flow has pathophysiological effects. ECs express several mechanoreceptors that respond to flow, but the mechanism for sensing shear stress direction is poorly understood. We determined, by using *in vitro* flow systems and magnetic tweezers, that β1 integrin is a key sensor of force direction because it is activated by unidirectional, but not bidirectional, shearing forces. β1 integrin activation by unidirectional force was amplified in ECs that were pre-sheared in the same direction, indicating that alignment and β1 integrin activity has a feedforward interaction, which is a hallmark of system stability. *En face* staining and EC-specific genetic deletion studies in the murine aorta revealed that β1 integrin is activated and is essential for EC alignment at sites of unidirectional flow but is not activated at sites of bidirectional flow. In summary, β1 integrin sensing of unidirectional force is a key mechanism for decoding blood flow mechanics to promote vascular homeostasis.

This article has an associated First Person interview with the first author of the paper.

## Abstract (precis)

**Summary:** By using flow systems, magnetic tweezers and knockout mice, we show that β1 integrin is activated by unidirectional hemodynamic shear force leading to Ca^2+^ signalling and cell alignment, but it is not activated by bidirectional force.

## INTRODUCTION

Although multiple mechanoreceptors have been identified, the fundamental mechanisms that cells use to sense the direction of force remain largely unknown. Arteries are exposed to mechanical forces of differing direction and magnitude via the action of flowing blood, which generates shear stress (mechanical drag) on the endothelial cells (ECs) that line the inner surface. Notably, atherosclerosis, a major cause of mortality in Western societies, develops at branches and bends of arteries that are exposed to disturbed non-uniform flow (Kwak et al., 2014). These flow fields are remarkably complex and include flows that oscillates in direction (bidirectional), secondary flows that are perpendicular to the main flow direction and low-velocity flows. By contrast, artery regions that are exposed to non-disturbed unidirectional shear stress are protected. The direction of shear stress has profound effects on EC physiology. Unidirectional shear stress induces EC alignment accompanied by quiescence, whereas bidirectional and other non-uniform shear stress profiles do not support alignment (Ajami et al., 2017; Feaver et al., 2013; Sorescu et al., 2004; Wang et al., 2013, 2006; Wu et al., 2011). ECs express several mechanoreceptors, including integrins, ion channels, the glycocalyx, primary cilia and G-protein-coupled receptors (Baeyens et al., 2014; Chen et al., 2015, 1999; Friedland et al., 2009; Givens and Tzima, 2016; Matthews et al., 2006; Shyy and Chien, 2002; Tzima et al., 2001, 2005). However, the mechanisms that allow cells to decode the direction of shear stress are poorly characterised and a key question in vascular biology.

The integrin family of α-integrin–β-integrin heterodimeric adhesion receptors mediate adhesion of cells to neighbouring cells or to the extracellular matrix (ECM) via interaction with specific ligands. This process involves quaternary structural changes in integrin heterodimers, whereby a low-affinity, bent configuration is converted into a high-affinity, extended form (Friedland et al., 2009; Li et al., 2017; Puklin-Faucher et al., 2006; Puklin-Faucher and Sheetz, 2009). The ability of integrins to sense and respond to force is essential for cell shape, tissue architecture, cell migration and other fundamental processes. In the vasculature, the influence of flow on EC physiology involves shear stress-mediated activation of integrins (Tzima et al., 2001), which engage with ECM thereby triggering outside-in signalling (Bhullar et al., 1998; Chen et al., 2015, 1999; Jalali et al., 2001; Orr et al., 2006, 2005; Shyy and Chien, 2002; Tzima et al., 2001). Activation of α5β1 integrins by shear stress leads to Ca^2+^ signalling (Buschmann et al., 2010; Loufrani et al., 2008; Matthews et al., 2006; Thodeti et al., 2009; Yang et al., 2011), which in turn regulates EC migration (Urbich et al., 2002) and inflammation (Bhullar et al., 1998; Luu et al., 2013; Orr et al., 2005; Chen et al., 2015; Budatha et al., 2018; Yun et al., 2016; Sun et al., 2016). One model for the role of integrins in shear stress signalling is that tension generated at the apical surface is transmitted through the cytoskeleton to integrins localised to the basal surface, thereby inducing structural changes that enhances their affinity for ECM ligands (Bhullar et al., 1998; Orr et al., 2006, 2005; Puklin-Faucher and Sheetz, 2009; Tzima et al., 2001). Recent studies using a chimeric version of α5 integrin in which the cytoplasmic domain was replaced with that from α2 integrin, revealed that flow drives ECM-dependent signalling through basally located α5 integrin to promote the inflammatory activation of ECs (Budatha et al., 2018; Yun et al., 2016). However, other studies have demonstrated that integrins localised to the apical surface of ECs can also respond to mechanical force (Conforti et al., 1992, 1991; Matthews et al., 2006), a finding that we have explored further in this paper.

Here, we studied the fundamental mechanism used by ECs to sense the direction of mechanical force. This topic has translational significance because the mechanoreceptors that sense unidirectional protective force could be potentially targeted therapeutically to treat atherosclerosis. Although there is abundant evidence for the role of β1 integrin in mechanotransduction, its potential role in sensing the direction of flow has not been studied previously. We concluded that β1 integrins are essential for EC sensing of force direction since they were activated by unidirectional force to drive EC alignment, but were not activated by bidirectional force. Thus β1 integrins are the first example of a receptor that is activated specifically by unidirectional flow.

## RESULTS

### β1 integrin is activated by unidirectional but not by bidirectional shearing force

**Fig. 1.**
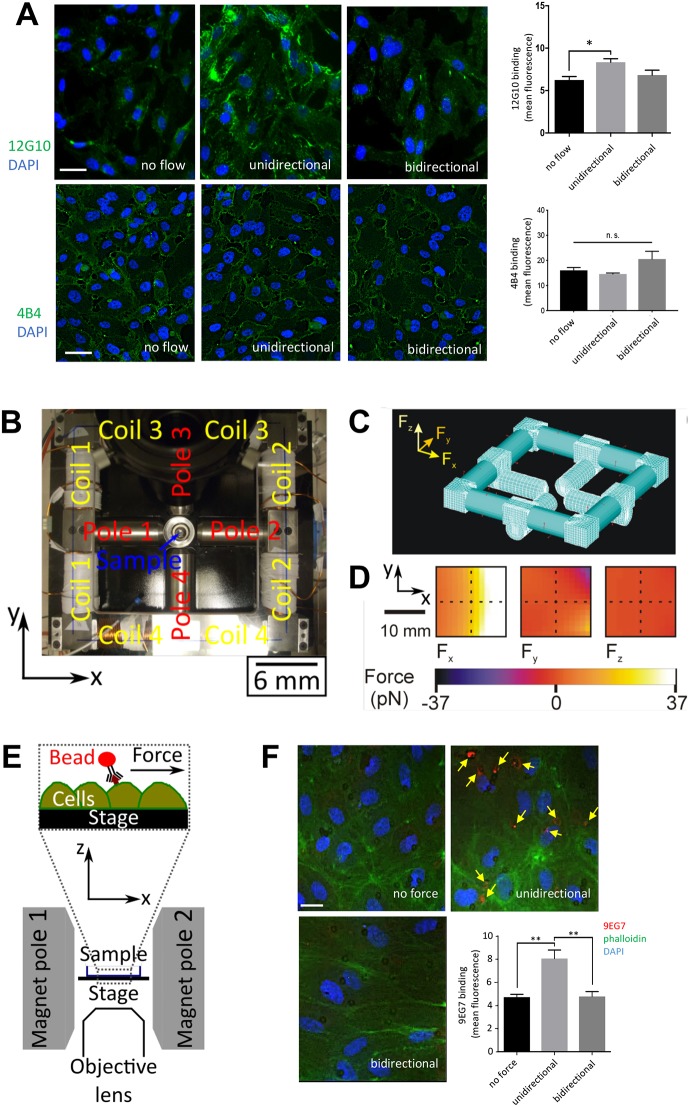
**β1 integrins are activated by unidirectional but not bidirectional shearing force.** (A) HUVECs were exposed to unidirectional or 1 Hz bidirectional flow for 3 min or remained under static conditions. Cells were stained with antibodies targeting active β1 integrins (12G10; green, upper images) or total β1 integrins (4B4; green, lower images) and DAPI (nuclei; blue). Representative images and 12G10 and 4B4 mean±s.e.m. fluorescence signal are shown. Scale bars: 10 μm. Data were pooled from five independent experiments. *P<0.05; n.s. not significant (one-way ANOVA with Tukey's test for multiple comparisons). (B) Photograph of the magnetic tweezers platform, showing which coil pairs activate which pole, the sample position and the coordinate system. (C) The finite element mesh used in the ANSYS model of the electromagnet is shown. (D) The modelled forces generated along the *x*-, *y*- and *z*-directions (*F_x_*, *F_y_* and *F_z_*, respectively) within the plane of the stage position when 10 A is applied to coil 2 are shown. The hatched lines indicate the imaging position. (E) A cross-section schematic diagram of the sample position, highlighting the direction of the applied force when pole 2 is activated. (F) 4B4-coated magnetic beads (targeting the βI domain of inactive β1 integrin) were incubated with HUVECs prior to the application of unidirectional or 1 Hz bidirectional force (∼16 pN) for 3 min. As a control, beads remained under no force. β1 integrin activation was quantified by immunostaining (9EG7; red, arrows) with co-staining of F-actin (phalloidin; green) and nuclei (DAPI; blue). Scale bar: 10 μm. Results are mean±s.e.m. and are pooled from four independent experiments. **P<0.01 (one-way ANOVA with Tukey's test for multiple comparisons).

To investigate whether β1 integrin responds to a specific flow direction, we exposed cultured human umbilical vein ECs (HUVECs) to shear stress (15 dyn/cm^2^) that was either unidirectional or bidirectional (1 Hz). Staining using 4B4 antibodies, which bind the total pool of β1 integrin, demonstrated that flow had no effect on β1 integrin expression (Fig. 1A, lower panels). By contrast, staining with the 12G10 antibody, which specifically binds to the high-affinity, extended β1 conformer, revealed that β1 integrins were activated by exposure to unidirectional but not bidirectional flow (Fig. 1A; upper panels). Super-resolution confocal microscopy demonstrated that the major portion of active β1 integrin was observed at the basal surface of ECs exposed to unidirectional flow and a minor portion localised to the apical surface (Fig. S1).

Since flow can alter the transport of materials as well as local mechanics, we used a magnetic tweezers platform to apply force directly to β1 integrins and determine whether force direction regulates their activity. An electromagnet was built in-house and coupled to a fluorescence microscopy platform fitted with an incubation chamber heated to 37°C, enabling live-cell imaging during operation of the tweezers. Passing current through copper coil pairs generated a magnetic field that was concentrated close to the sample by the corresponding pole piece. In this study, poles 1 and 2 were used to generate unidirectional or bidirectional forces (poles 3 and 4 were not used; Fig. 1B). Computational modelling revealed that the force generated at the centre of the imaging area of the microscope was 16 pN, with a 7.5% (1 pN) variation across the imaging area [see Materials and Methods, Eqn (1)]. Forces along the *y*- and *z*-directions were negligible (<0.1% of the total force), so the force generated by the tweezers was directed almost entirely along the *x*-direction, towards the activated pole (Fig. 1C,D). As the force was parallel to the stage, it mimicked the shearing action experienced by receptors under flow (Fig. 1E). The generation of force was validated by observing the movements of suspended paramagnetic beads (see, for example, Movie 1).

The influence of force direction on β1 integrin activation was assessed by applying superparamagnetic beads coated with antibodies that target the βI domain of the inactive β1 conformer (4B4 antibodies) to the apical surface of HUVECs prior to the application of unidirectional or bidirectional forces. After 3 min force, β1 integrin activation was quantified by staining using 9EG7 antibodies, which specifically recognise the extended high-affinity conformer (Byron et al., 2009; Mould et al., 1995; Su et al., 2016) and bind to a portion of β1 integrin that is not recognised by 4B4 (Bazzoni et al., 1995). Since the shear stress generated by the bead is given by the force per contact area, we estimate the bead produces between 10–15 dyn/cm^2^, assuming that between a quarter and a sixth of the surface area of the bead is in contact with the cell. This is a comparable magnitude to the shear stress in human arteries (Kwak et al., 2014). We found that the application of unidirectional force enhanced 9EG7 binding, indicating that mechanical activation of β1 integrin is induced by unidirectional force, whereas bidirectional force had no effect (Fig. 1F). Therefore, unidirectional force converts β1 integrin into an extended high-affinity conformer whereas bidirectional force does not.

### The βI domain of β1 integrin senses unidirectional force

**Fig. 2.**
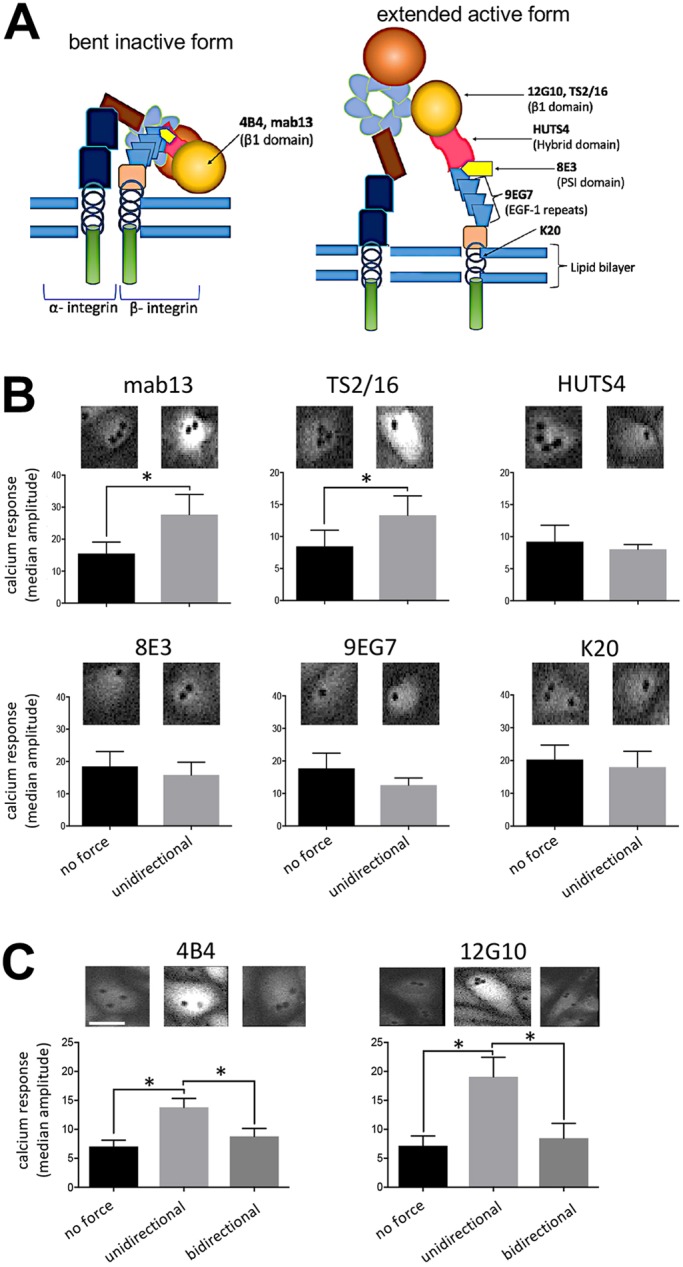
**β1 integrins sense unidirectional force via the βI domain.** (A) Schematic representation of domains and antibody binding sites on the bent, inactive or extended, active β1 integrin. (B) HUVECs were loaded with Cal-520 and then incubated with beads coated with mab13, TS2/16, HUTS4, 8E3, 9EG7 or K20 antibodies. Beads were exposed to unidirectional force (∼16 pN) or no force as a control. (C) HUVECs were loaded with Cal-520 and then incubated with beads coated with antibodies targeting inactive (4B4) or active (12G10) β1 integrins. Beads were exposed to unidirectional force (∼16 pN), bidirectional force (1 Hz ∼16 pN) or no force. (B,C) Ca^2+^ responses were recorded for 3 min using fluorescence microscopy. Representative images are shown. Data were pooled from five independent experiments and the median amplitude of the first peak of the Ca^2+^ response was calculated and is presented as the means±s.e.m. *P<0.05 [two-tailed paired Student's *t*-test (B) or one-way ANOVA test, with Tukey's test for multiple comparisons (C)].

To determine the regions of β1 integrin that are responsible for force sensing, we applied force using monoclonal antibodies that target specific domains (Byron et al., 2009) (Fig. 2A). Activation of β1 integrin by mechanical force is known to induce Ca^2+^ signalling (Matthews et al., 2006), and we therefore used Ca^2+^ accumulation as a readout, as determined by using the fluorescent Ca^2+^ reporter (Cal-520). Force did not cause detachment of beads from cells in these experiments (Fig. S2A). The application of unidirectional force to the βI domain of the inactive form (via mab13 or 4B4 antibodies) or to the active extended form (via TS2/16 or 12G10 antibodies) induced Ca^2+^ accumulation (Fig. 2B,C), whereas force application to the hybrid (HUTS4), PSI (8E3), EGF-like (9EG7) domains or membrane-proximal region (K20) had no effect (Fig. 2B). Of note, applying force to the βI domain discriminated between different patterns of force because it induced Ca^2+^ signalling in response to unidirectional but not bidirectional force (Fig. 2C). As a control, it was demonstrated that the application of force to poly-D-lysine-coated beads, which bind negatively charged molecules, had no effect on Ca^2+^ levels (Fig. S2B). Thus, it was concluded that the βI domain of β1 integrin is a sensor of force direction; it responds specifically to unidirectional force leading to activation of β1 integrin and downstream Ca^2+^ signalling. Since 4B4 is an inhibitory antibody that prevents integrin extension, ligand binding is by definition not required for the effect. Moreover, our observation that force can promote signalling when applied to pre-activated extended forms of β1 integrin implies that tension is transmitted through β1 integrin to the cell during signal transduction.

**Fig. 3.**
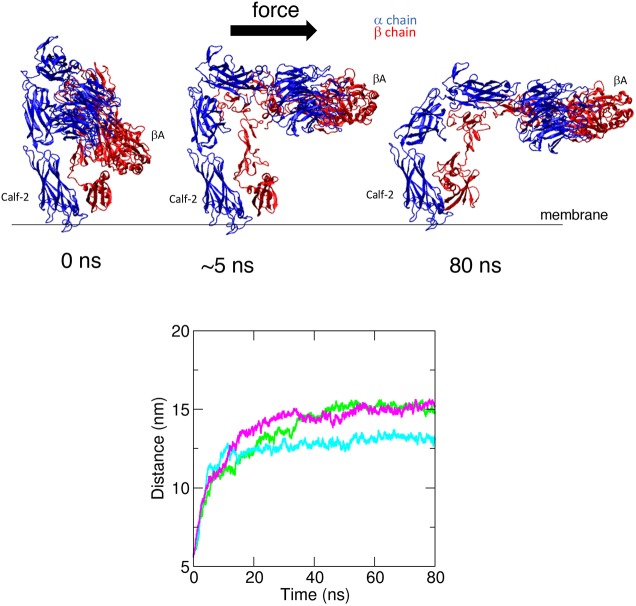
**SMD simulations of integrin structural rearrangements in response to mechanical force.** SMD simulations were performed using the αVβ3 integrin ectodomain. A force (200 kJ mol^−1^ nm^−1^) parallel to the membrane was applied on the βA domain of the head region of the inactive form. The constant application of force triggered intramolecular structural rearrangements and extension of the molecule in three independent simulations. The structure is shown at the beginning of the simulations and after the application of force for 5 ns or 80 ns. αV is shown in blue and β3 integrin is shown in red (upper panels). Structural rearrangements were quantified by measuring the distance between the Calf-2 and βA domains over the course of the simulation (lower panels). The coloured lines represent the three repeat simulations. Force consistently induced extension of the integrin heterodimer.

The mechanism of integrin activation by shear stress was also studied through steered molecular dynamic (SMD) simulations. A 3D structure of the β1 integrin ectodomain is not available and therefore we focussed on the ectodomain structure of αVβ3 (PDB 3IJE; Xiong et al., 2009). We predicted that the mechanism of integrin activation by shear stress will be conserved between the β3 and β1 subunits because they have a high structural similarity and a similar fold in the inactive state. Consistent with this, magnetic tweezer experiments demonstrated that β3 integrin signalling was activated by unidirectional but not bidirectional force (Fig. S3), which is similar to observations made for β1 integrin (Fig. 2). Therefore, we can extrapolate SMD simulations carried out using β3 integrin to β1 integrin. Previous SMD simulations have demonstrated that a pulling force applied tangentially to the membrane induces integrin activation by unfolding and increases the angle of the βI/hybrid domain hinge, subsequently leading to αβ leg separation (Chen et al., 2012; Puklin-Faucher et al., 2006). However, to mimic the effects of shear stress, we applied force (200 kJ mol^−1^ nm^−1^) in parallel to the membrane to the βA domain and demonstrated that it converted the bent inactive form into an extended form that was tilted relative to the membrane (Fig. 3; Movie 2). Owing to the size of this system (∼1.5 M atoms), we have used forces that are higher than physiological levels. For this reason, our simulations cannot provide information about the timescale of integrin activation by shear stress; however, they support our magnetic tweezers data showing that force applied parallel to the membrane can cause integrin activation.

In conclusion, we propose that the application of force parallel to the membrane to the βI domain of the bent inactive form elicits extension of the molecule, which is required for Ca^2+^ signalling and this is supported by the SMD simulation data.

### β1 integrin elicits Ca^2+^ signalling in response to unidirectional flow but not bidirectional flow

**Fig. 4.**
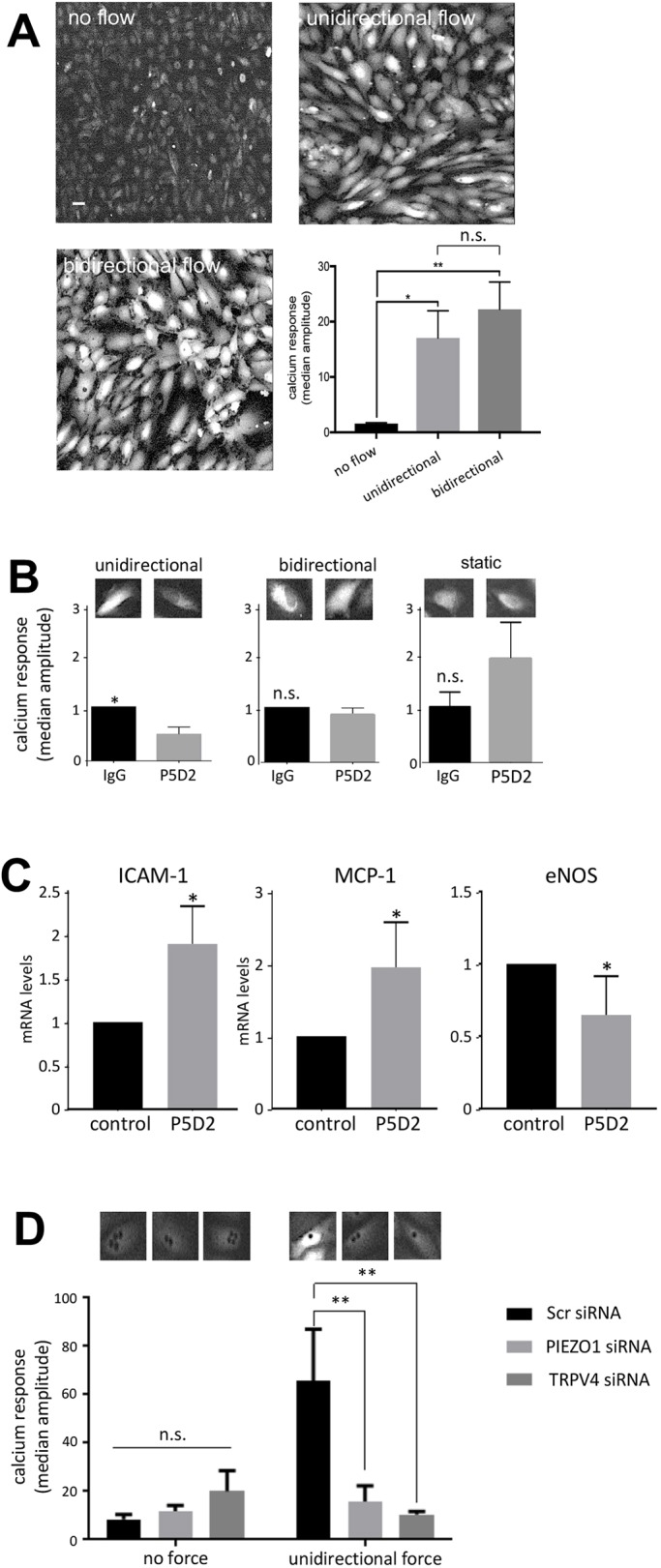
**β1 integrins induce Ca^2+^ accumulation in response to unidirectional flow via Piezo1 and TRPV4.** (A) HUVECs were loaded with the Ca^2+^ fluorescent dye (Cal-520), then exposed to unidirectional or 1 Hz bidirectional flow and Ca^2+^ responses were recorded for 3 min using fluorescence microscopy. Representative images are shown. Scale bar: 10 μm. (B) HUVECs were loaded with Cal-520 and then incubated with P5D2 (β1 integrin-blocking antibody) or with total isotype-matched mouse IgG as a control. They were then exposed to unidirectional or 1 Hz bidirectional flow or static conditions and Ca^2+^ responses were recorded for 3 min. (C) HUVECs were incubated with P5D2 or with total isotype-matched mouse IgG as a control. They were then exposed to unidirectional flow for 72 h prior to quantification levels of mRNAs encoding ICAM-1, MCP-1 or eNOS by qRT-PCR. (D) HUVECs were transfected with siRNA targeting Piezo1, TRPV4 or with scrambled (Scr) sequences, as a control. After 72 h, cells were loaded with Cal-520 and then incubated with beads coated with 12G10 antibodies, targeting active β1 integrins. Beads were exposed to unidirectional force (∼16 pN) or no force. Ca^2+^ responses were recorded for 3 min. The bar graphs in each panel show data pooled from five (A), six (B), three (C) or four (D) independent experiments. For A, B and D, the median amplitude of the first peak of the Ca^2+^ response was calculated and is presented as the means±s.e.m. *P<0.05, **P<0.01 [one-way ANOVA test, with Tukey's test for multiple comparisons (A), a two-tailed paired Student's *t*-test (B,C) or a two-way ANOVA (D)].

Next, we investigated the mechanism by which β1 integrin converts unidirectional flow into Ca^2+^ signalling as this pathway is a pivotal regulator of EC physiology (Ando and Yamamoto, 2013). Imaging of HUVECs loaded with Cal-520 revealed Ca^2+^ accumulation in the cytosol in response to unidirectional or bidirectional flow (Fig. 4A; Movie 3), indicating that both of these flow patterns drive Ca^2+^ signalling. The potential role of β1 integrin was tested using P5D2 inhibitory antibodies that bind close to the ligand-binding pocket and induce conformational changes that reduce ligand binding affinity and displace the conformational equilibrium of β1 integrin towards the inactive, non-signalling, form. Pre-treatment with inhibitory P5D2 antibodies reduced Ca^2+^ accumulation in response to unidirectional flow but not bidirectional flow or static conditions (Fig. 4B). Thus, although both unidirectional and bidirectional flow activate Ca^2+^ signalling, β1 integrin is specifically required for the response to unidirectional flow. We next investigated the role of β1 integrin in transcriptional responses to flow, and observed that treatment with P5D2 enhanced the expression of the inflammatory ICAM-1 and MCP-1 (also known as CCL2) and simultaneously reduced the expression of eNOS (also known as NOS3) (Fig. 4C). Thus, β1 integrin activation is required for eNOS induction and suppression of inflammatory gene expression in response to unidirectional flow.

We next determined whether β1 integrin-dependent Ca^2+^ signalling involves Piezo1 and TRPV4, since these Ca^2+^-permeable channels are known to sense shear stress (Köhler et al., 2006; Mendoza et al., 2010; Nguyen et al., 2014; Thodeti et al., 2009). HUVECs were treated with specific siRNAs to silence Piezo1 or TRPV4 (Fig. S4A) prior to the application of unidirectional force via magnetic tweezers coupled to 12G10-coated superparamagnetic beads. Silencing of Piezo1 or TRPV4 significantly reduced the accumulation of Ca^2+^ (Fig. 4D) in HUVECs exposed to unidirectional flow, indicating that both channels are involved in Ca^2+^ signalling. Consistent with this, β1 integrin-dependent Ca^2+^ signalling was significantly reduced upon treatment with EGTA, indicating a requirement for extracellular Ca^2+^ (Fig. S5). By contrast, the response to bidirectional flow was only partially reduced upon treatment with EGTA, and this difference was not statistically significant. However, silencing of Piezo1 or TRPV4 did not influence β1 integrin signalling in response to unidirectional flow (Fig. S4B), indicating that these channels do not act upstream of β1 integrin. We conclude that unidirectional force induces Ca^2+^ signalling via a mechanism that requires β1 integrin-mediated activation of Piezo1 and TRPV4 coupled to extracellular Ca^2+^, whereas bidirectional force signals via a β1 integrin-independent mechanism.

### Unidirectional shear stress induces a feedforward interaction between β1 integrin activation and cell alignment

**Fig. 5.**
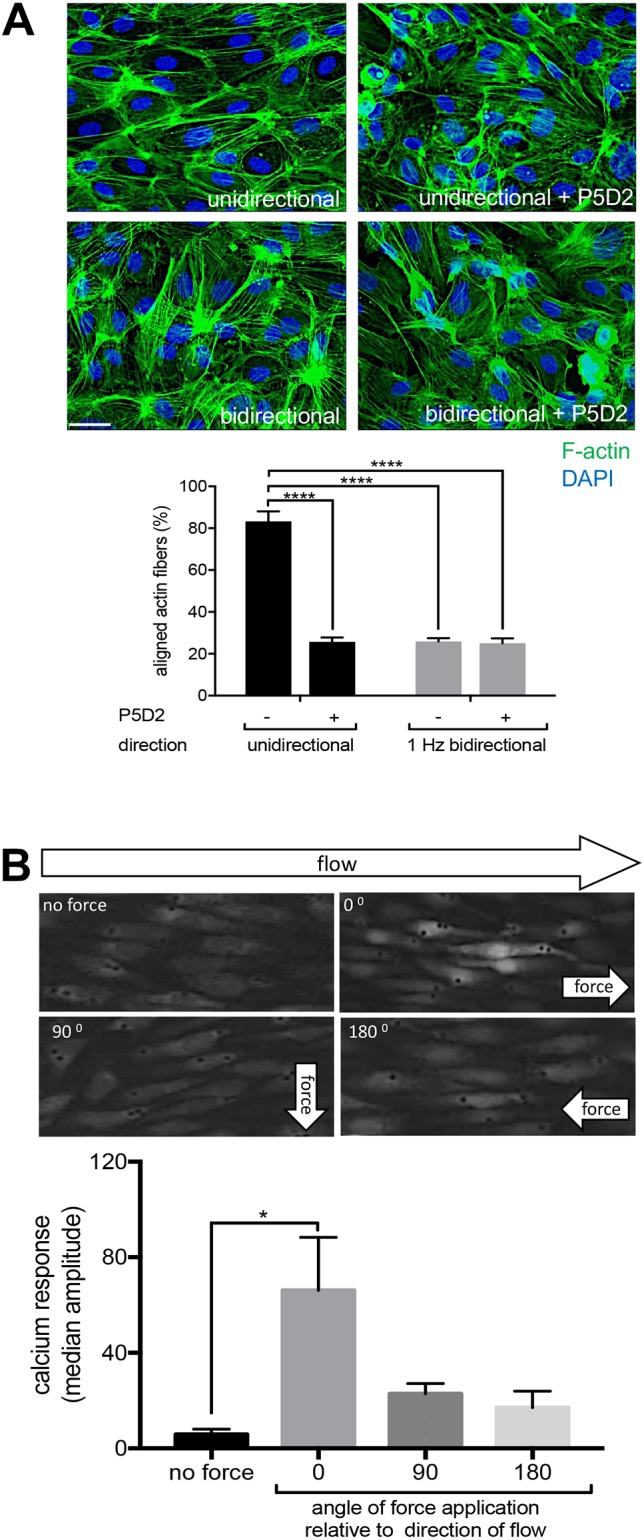
**β1 integrin activation and cell alignment response have a feedforward interaction under unidirectional flow.** (A) HUVECs pre-treated under static conditions with P5D2 (β1 integrin-blocking antibody) or control IgG were exposed to unidirectional or 1 Hz bidirectional flow for 24 h and then stained with FITC–phalloidin (green; actin fibres) or DAPI (blue; nuclei). The means±s.e.m. proportion (%) of cells with actin fibres aligned with the major cell axis (within 30°) was calculated. Scale bar: 10 μm. (B) HUVECs were exposed to unidirectional flow for 72 h and then maintained under static conditions. They were loaded with the Ca^2+^ fluorescent dye Cal-520 and incubated with beads coated with 12G10 antibody. Unidirectional force was then applied either in the same direction as the pre-shearing (0°), or tangentially (90°) or in the opposite direction (180°). Ca^2+^ responses were recorded for 3 min using fluorescence microscopy. Representative images are shown. The bar graphs in each panel show data were pooled from three independent experiments. The median amplitude of the first peak of the Ca^2+^ response was calculated and is presented as the mean±s.e.m. *P<0.05, ****P<0.0001 [two-way ANOVA test (A) or a one-way ANOVA test with Tukey's test for multiple comparsions (B)].

Ca^2+^ signalling induces alignment of ECs in the direction of flow, which is essential for vascular homeostasis (Wang et al., 2013). To investigate the role of β1 integrins in this process, we treated HUVECs with P5D2 activity blocking antibodies (or with non-binding antibodies as a control) during exposure to unidirectional or bidirectional flow. ECs aligned specifically in response to unidirectional flow and this was blocked by P5D2, demonstrating an essential role for β1 integrin activation in this process (Fig. 5A). Since EC polarity alters their response to flow (Wang et al., 2013), we investigated whether EC alignment could influence β1 integrin sensing of mechanical force. This was tested by exposing ECs to shear stress and subsequently measuring the effects of applying force through β1 integrin either in the same direction as the flow, or in the opposite direction or perpendicular to the direction of flow. We observed an anisotropic response, with faster signalling when force was applied in the same direction as the flow, and slower responses when force was applied in the opposite direction or tangentially (Fig. 5B). Thus, unidirectional force sensing by β1 integrins is enhanced in cells that are aligned with flow, indicating a feedforward interaction between β1 integrin activation and cell alignment.

### β1 integrin is essential for EC alignment at sites of unidirectional shear *in vivo*

**Fig. 6.**
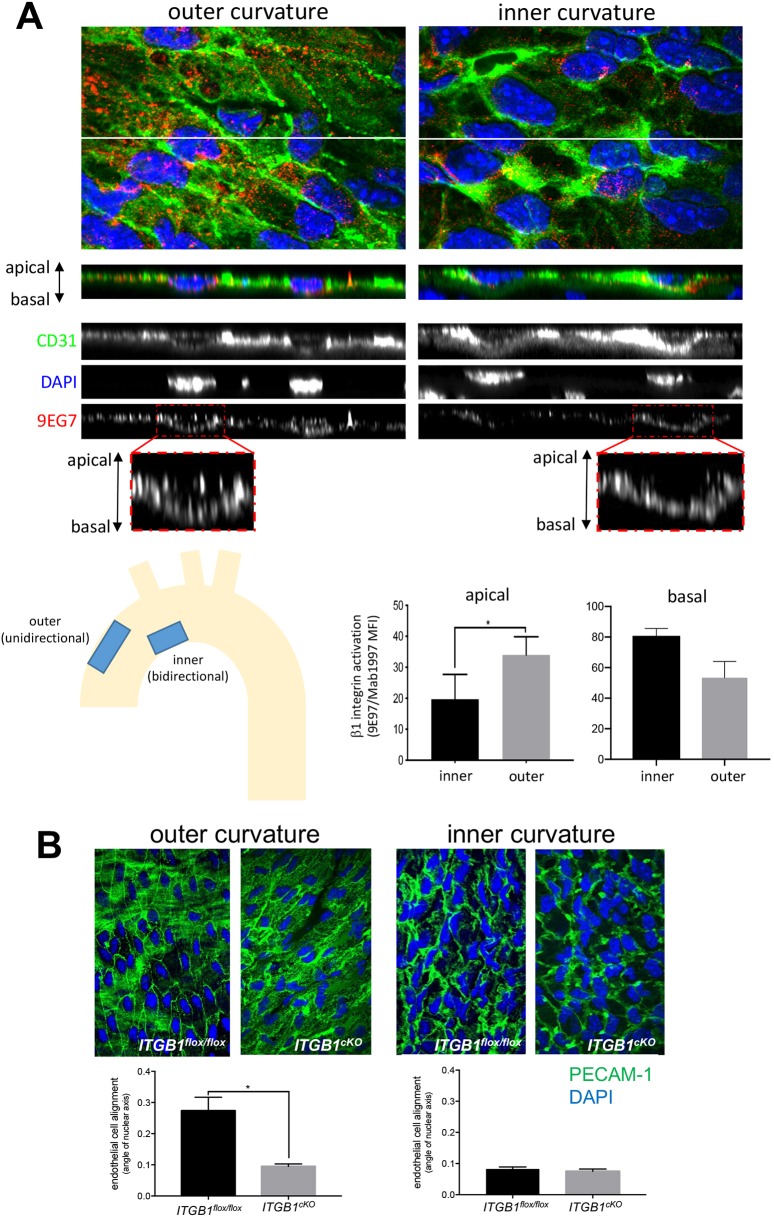
**β1 integrin activation is essential for EC alignment at sites of unidirectional flow *in vivo*.** (A) Mouse aortic arches were stained *en face* with antibodies targeting active β1 integrins (9EG7; red). ECs were co-stained using anti-PECAM-1 antibodies (green) and nuclei were counterstained using DAPI (blue). Fluorescence was measured at the outer curvature (unidirectional flow) and inner curvature (bidirectional flow) regions after super-resolution confocal microscopy. Representative *z*-series stacks of images are shown, and apical and basal surfaces are indicated. Note that 9EG7 stained apical and basal surfaces at the outer curvature, but was restricted to the basal side at the inner curvature. Levels of active β1 integrins at the apical and basal surfaces were calculated by quantifying the ratio of fluorescence from 9EG7 (active form) and Mab1997 (total β1 integrin). Results are mean±s.e.m. (*n*=3). (B) EC alignment was quantified at the outer curvature (unidirectional flow) and inner curvature (disturbed bidirectional flow) of the aortic arch by *en face* staining of PECAM-1 (green) and DAPI (nuclei; blue) in *Itgb1^flox/flox^* or *itgb1*^cKO^ mice (*n*=3). EC alignment was quantified by measuring the angle of the major axis of the nucleus. Mean±s.e.m. fluorescence values are shown (*n*=3 mice). *P<0.05 (two-tailed paired Student's *t*-test).

To assess whether β1 integrin activation correlates with flow direction *in vivo*, we studied precise locations within the murine aortic arch that were previously shown by computational fluid dynamic studies to be exposed to high unidirectional flow (outer curvature) or to low velocity flow that oscillates in direction (bidirectional flow) (Suo et al., 2007). *En face* staining was performed followed by super-resolution confocal microscopy to quantify the level of β1 integrin at apical and basal surfaces. Quantification of the total pool of β1 integrin (using Mab1997 antibody) revealed that the majority of this protein localised to the basal surface, but that a proportion was also detected at the apical surface at both the inner and outer curvatures (Fig. S6A). By contrast, staining using 9EG7 antibodies revealed that active β1 integrin localised to the apical surface of the outer curvature, but was not observed at the inner curvature, whereas active β1 integrin at the basal surface was observed at both regions (Fig. 6A; Movie 4). Thus, β1 integrin activation (calculated as a ratio of 9EG7 to Mab1997 fluorescence) at the apical surface was significantly higher at the outer compared to the inner curvature, whereas β1 integrin activation at the basal surface did not vary according to anatomy (Fig. 6A; Movie 4). These data are consistent with the finding that β1 integrin is activated exclusively by unidirectional shear stress in cultured EC (Figs 1 and 2).

To study the function of β1 integrin *in vivo*, we deleted it conditionally from adult EC noting that deletion from embryonic EC is lethal (Carlson et al., 2008; Lei et al., 2008; Tanjore et al., 2008; Zovein et al., 2010). Conditional genetic deletion of β1 integrin (*Itgb1*) from ECs was confirmed by *en face* staining using anti-β1 integrin antibodies (Fig. S6B). Deletion of β1 integrin resulted in a significantly reduced EC alignment at the outer curvature (unidirectional flow) but did not alter EC morphology at the inner curvature of the murine aortic arch (disturbed bidirectional flow), which showed non-aligned cells in both wild-type and β1 integrin conditional knockout mice (Fig. 6B). It should be noted that the β1 integrin conditional knockout does not discriminate between apical and basal pools of integrin; however it can be used to support the concept that β1 integrin responds specifically to unidirectional flow. Collectively, our data demonstrate that β1 integrin activation by unidirectional shear stress is an essential driver of EC alignment.

## DISCUSSION

### Endothelial sensing of flow direction – the role of β1 integrins

The ability of ECs to sense the direction of blood flow is essential for vascular health and disease (Wang et al., 2013). It underlies the focal distribution of atherosclerotic lesions, which develop at parts of arteries that are exposed to complex flow patterns including bidirectional flow but does not develop at sites of unidirectional flow. It is well established that ECs sense the shearing force generated by flow via multiple mechanoreceptors including the VE-cadherin–PECAM-1–VEGFR2 trimolecular complex (Tzima et al., 2005), Piezo1 (Li et al., 2014) and several others. However, the molecular mechanisms that convert directional cues into specific downstream responses are poorly understood. Recent studies have indicated that PECAM-1 can sense both unidirectional and disturbed flow leading to the transmission of protective and inflammatory signals accordingly. Thus, PECAM-1 knockouts have a fascinating phenotype characterised by enhanced lesions at sites of unidirectional flow and reduced lesion formation at sites of disturbed flow (Goel et al., 2008; Harry et al., 2008). On the other hand, the transmembrane heparan sulphate proteoglycan syndecan-4 is required for EC alignment under shear stress but is dispensable for other mechanoresponses, indicating a role in sensing of flow direction (Baeyens et al., 2014).

Here, we conclude that β1 integrins are sensors of force direction through the following lines of evidence. First, β1 integrin converts from a bent inactive form into an extended active conformer in response to unidirectional but not bidirectional shearing force. Second, SMD simulations revealed that force applied parallel to the membrane can cause structural rearrangements leading to β1 integrin extension. Third, unidirectional shearing force induces Ca^2+^ signalling via a β1 integrin-dependent mechanism whereas the response to bidirectional force is independent from β1 integrin. Fourth, silencing of β1 integrin prevented alignment of cultured ECs exposed to unidirectional shear stress but did not alter the morphology of cells exposed to bidirectional shear. Fifth, β1 integrin was activated specifically at sites of unidirectional shear stress in the murine aorta, and, finally, deletion of β1 integrin from EC reduced EC alignment at sites of unidirectional shear stress in the murine aorta but did not alter morphology at sites of disturbed flow.

### Is β1 integrin a direct sensor of flow?

There is abundant evidence that integrins can respond to flow indirectly via signals elicited from mechanoreceptors including PECAM-1 (Collins et al., 2012) and Piezo1 (Albarrán-Juárez et al., 2018). Thus, flow causes activation of integrins on the basal surface of ECs, which subsequently engage with ligand and trigger outside-in signalling (Orr et al., 2006, 2005; Tzima et al., 2001). However, our observations suggest that there is an apical pool of β1 integrin that is activated by unidirectional shear stress to induce downstream signalling and cell alignment. Our data are consistent with a previous study in which mechanical signalling of apical integrins was induced with magnetic tweezers (Matthews et al., 2006). They also resonate with biochemical and electron microscopy studies that detected β1 integrin and other integrins at the apical surface of ECs (Conforti et al., 1992, 1991). However, they contrast with other studies that detected basal but not apical pools of β1 integrin using confocal microscopy (Li et al., 1997; Tzima et al., 2001). The reason for this discrepancy is uncertain, but may relate to our use of super-resolution microscopy, which can delineate apical and basal surfaces of EC (<1 µm depth) more accurately than conventional confocal microscopy techniques. It is important to note that our observations showing that apical β1 integrin can respond to force does not preclude the important and well-established role for basally located integrins, and we suggest that both pools contribute to flow sensing. Indeed, it is plausible that the function of α5β1 heterodimers varies according to their localisation on basal or apical surfaces since basally located integrin is activated in response to disturbed flow (Sun et al., 2016; Albarr-Juárez et al., 2018), whereas we found that apically located integrin is activated exclusively by unidirectional flow. The mechanisms that ECs use to integrate these divergent downstream signals from apical and basal pools of β1 integrin should now be investigated further.

Fluid dynamics predict that blood flow will approach zero velocity near to the vessel wall (no-slip condition) and therefore it is uncertain how proteins at the apical surface are activated by shear stress. However, ECs possess structures that project into the lumen that may be important for mechanosensing, including the primary cilium and the glycocalyx, which is a layer of glycolipids, glycoproteins and proteoglycans at the apical surface of ECd. Although the glycocalyx is often absent from cultured ECs (Chappell et al., 2009), it has been observed on arterial endothelium where it can transmit shear forces to the apical surface of endothelial cells (Bartosch et al., 2017). We observed that β1 integrin was activated on the apical surface of murine aortic endothelium and it would be interesting in future studies to assess whether glycosylation of β1 integrin (Xu et al., 2018) and the glycocalyx are regulators of this process.

### Mechanism of unidirectional flow sensing and cell alignment

**Fig. 7.**
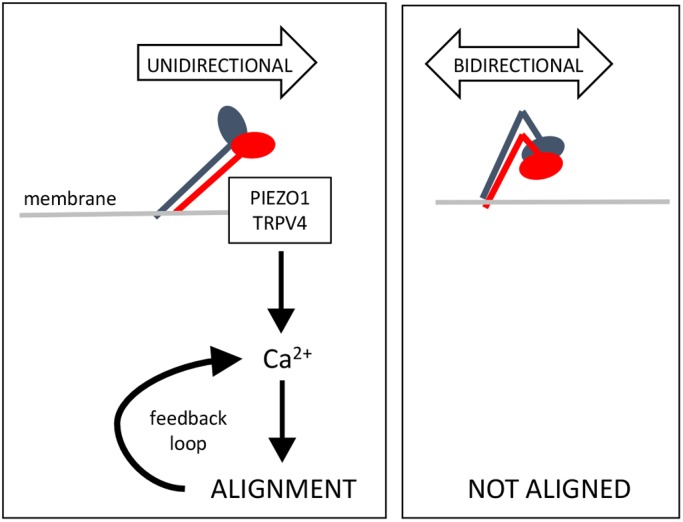
**Model to explain the mechanism of β1 integrin sensing of flow direction.** Unidirectional flow (left) induces structural changes in the ectodomain of β1 integrins causing them to extend. The integrin subsequently acts as a ‘sea anchor’ thereby inducing the accumulation of Ca^2+^ via Piezo1 and TRPV4 leading to cell alignment. This forms a feedforward loop to enhance Ca^2+^ signalling therefore promoting physiological stability. Under bidirectional flow (right), β1 integrin is not activated.

By using magnetic tweezers, we determined that unidirectional shearing force induces downstream signals via a two-stage process. First, it converts the bent inactive form of β1 integrin into an extended form. Second, the extended form of β1 integrin transmits force to the cell to elicit downstream signalling. At the basal surface of cells, β1 integrin is anchored to extracellular matrix and therefore can transmit tension to the cell (Friedland et al., 2009; Nordenfelt et al., 2016; Zhu et al., 2008). However, we observed that apical β1 integrin can sense force in the absence of ligand binding. Although apical β1 integrins are not anchored to extracellular matrix, we hypothesise that they may function as a ‘sea anchor’ in cells exposed to flow, thereby allowing force to be transmitted to the cell. Thus, we propose that unidirectional shear stress induces tension in β1 integrin leading to downstream signalling whereas bidirectional shear stress is insufficient because it switches direction before tension can be established (Fig. 7). Our model is consistent with other studies that demonstrated that mechanical forces can activate β1 integrins independently from ligand binding (Ferraris et al., 2014; Petridou and Skourides, 2016).

We observed that β1 integrin sensing of unidirectional force induced Ca^2+^ accumulation via Piezo1 and TRPV4. These data are consistent with the known roles of Piezo1 (Li et al., 2014) and TRPV4 (Köhler et al., 2006) in shear sensing and with a previous report of crosstalk between β1 integrin and TRPV4 in endothelium subjected to shear stress (Matthews et al., 2010). Our study also revealed that β1 integrins are essential for alignment of EC under unidirectional shear stress. Since Piezo1 and Ca^2+^ positively regulate EC alignment (Li et al., 2014; Miyazaki et al., 2007), we propose that unidirectional force induces EC alignment via β1 integrin, Piezo1 and TRPV4-dependent Ca^2+^ signalling. It should be noted however that β1 integrin has pleiotropic functions in EC and therefore the effects of knockdown or deletion of β1 integrin could be downstream from the flow sensing mechanism that we propose. Therefore, the mechanisms of crosstalk between β1 integrins, Piezo1 and TRPV4 during endothelial responses to mechanical force and their integration with cell alignment should be studied further.

Interestingly, we observed that direction-specific β1 integrin signalling is anisotropic because it is enhanced in cells that are pre-aligned in the direction of force application but reduced in cells exposed to flow in the opposite direction or tangentially. The mechanism of anisotropy is uncertain but would be expected to involve mechanisms that limit rotational diffusion and maintain the orientation of β1 integrin. It is plausible that the mechanism involves flow-mediated alteration of actin dynamics (Nordenfelt et al., 2017) or membrane fluidity (Sun et al., 2016), which are known to modulate integrin orientation and activation. Since β1 integrin drives EC alignment and vice versa, we conclude that a feedforward loop exists between β1 integrin activation and alignment. Feedforward systems are intrinsic to physiological stability and therefore the positive interaction between EC alignment and β1 mechanosensing is predicted to maintain long-term vascular homeostasis at sites of unidirectional flow.

### Significance of our study

Our study provides insight into the mechanisms that ECs use to decode complex mechanical environments to produce appropriate physiological responses. Focussing on β1 integrin, we found that it is a specific sensor of unidirectional flow driving downstream signalling and EC alignment. These findings suggest the exciting possibility that specific mechanical force profiles are sensed by specific mechanically cognate receptors to elicit distinct downstream responses. Future studies should now identify the mechanoreceptors that sense other mechanical forces profiles, for example, bidirectional force. Our observation that EC responses to distinct force profiles can be modified by targeting specific mechanoreceptors has implications for the treatment of atherosclerosis, which develops and progresses at sites of disturbed flow (Kwak et al., 2014).

## MATERIALS AND METHODS

### Antibodies

Several monoclonal antibodies that recognise β1 integrin were purchased from commercial sources: 12G10 (Abcam, ab30394; specifically recognises the active form), 9EG7 (BD Pharmingen, 553715; specifically recognises the active form), P5D2 (Abcam, ab24693; blocks activation), 4B4 (Beckman Coulter, 41116015), Mab1997 (Merck, Mab1997; MB1.2). Several monoclonal antibodies that recognise β1 integrin (TS2/16, HUTS4, 8E3, K20, mab13) were generated in-house from hybridoma (Byron et al., 2009; Mould et al., 1995). Rabbit anti-integrin β1 antibodies (EPR16895; Abcam, ab179471), and antibodies recognising murine CD31 (MEC13.3; BioLegend) and human CD144 (55-7H1; BD Biosciences, 555661) were obtained commercially.

### EC culture and application of shear stress

HUVECs were isolated through collagenase digestion and maintained in M199 growth medium supplemented with fetal bovine serum (20%), L- glutamine (4 mmol l^−1^), endothelial cell growth supplement (30 μg ml^−1^), penicillin (100 U μl^−1^), streptomycin (100 μg ml^−1^) and heparin (10 U ml^−1^). HUVECs (25×10^4^) were seeded onto 0.4 mm microslides (Luer ibiTreat, ibidi™) pre-coated with 1% fibronectin (Sigma) and used when they were fully confluent. Chamber slides were placed on the stage of an inverted light microscope (Nikon^®^ Eclipse Ti) enclosed in a Perspex box pre-warmed to 37°C. Unidirectional or 1 Hz bidirectional flow of 15 dynes cm^−2^ for the indicated time was applied using the ibidi™ syringe pump system. Pharmacological inhibition of β1 integrin activation was performed using 1–10 μg ml^−1^ P5D2 antibody (Abcam).

### Gene silencing and quantitative RT-PCR

Gene silencing was performed by using siRNA sequences from Dharmacon [targeting Piezo1 (L-020870-03) or TRPV4 (L-004195-00)]. A non-targeting control siRNA (D-001810-10) was used as a control. HUVECs were transfected by using the Neon transfection system (Invitrogen) and following the manufacturer's instructions. The final siRNA concentration was 50 nM. To determine the efficiency of the knockdown, total RNA was extracted using RNeasy Mini kit (QIAGEN) according to manufacturer's protocol and 500 ng of total RNA was subjected to cDNA synthesis using an iScript reverse transcriptase (Bio-Rad). The resulting cDNA was used as a template for quantitative RT-PCR (qRT-PCR) using gene-specific primers and SsoAdvanced Universal SYBR Green Supermix from Bio-Rad. Amplification of the housekeeping gene *HPRT1* was used as an internal control. The following primer sequences were used: *HPRT1*, forward 5′-TTGGTCAGGCAGTATAATCC-3′ and reverse 5′-GGGCATATCCTACAACAAAC-3′; *PIEZO1*, forward 5′-GCCGTCACTGAGAGGATGTT-3′ and reverse 5′-ACAGGGCGAAGTAGATGCAC-3′; *TRPV4*, forward 5′-CTACGGCACCTATCGTCACC-3′ and reverse 5′-CTGCGGCTGCTTCTCTATGA-3′; *eNOS*, forward 5′-TGAAGCACCTGGAGAATGAG-3′ and reverse 5′-TTGACCATCTCCTGATGGAA-3′; *MCP-1*, forward 5′-GCAGAAGTGGGTTCAGGATT-3′ and reverse 5′-TGGGTTGTGGAGTGAGTGTT-3′; *ICAM-1*, forward 5′-AACCAGAGCCAGGAGACACT-3′ and reverse 5′-TCTGGCTTCGTCAGAATCAC-3′.

### Immunofluorescence staining of cultured ECs

Activation of β1 integrin was assessed by immunofluorescence staining using 9EG7 or 12G10 antibodies (both at 1:100) and Alexa Fluor 488-conjugated secondary antibodies (Invitrogen). Imaging was performed using a fluorescence microscope (Olympus) or a super-resolution confocal microscope (Zeiss LSM880 AiryScan Confocal).

### Quantification of Ca^2+^ responses

HUVECs seeded onto 1% fibronectin-coated 0.4 microslides (Luer ibiTreat, ibidi™) or 35 mm microdishes (µ-Dish 35 mm, ibidi™) were incubated with 50 μg Cal-520™ (AAT Bioquest^®^) and Pluronic^®^ F-127 (Invitrogen). For testing the effect of the directionality of force on pre-aligned cells, HUVECs were seeded onto six-well plates with circular glass coverslips (13 mm diameter) attached to the periphery of the wells. The cells were exposed to flow for 72 h using the orbital shaker model (Warboys et al., 2014), and the coverslips subsequently removed, placed into ibidi 35 mm microdishes and incubated with Cal520™ and Pluronic^®^ F-127 as described above. After incubation, cells were washed twice with HEPES-buffered saline Ca^2+^-containing media (134.3 mM NaCl, 5 mM KCl, 1.2 mM MgCl_2_, 1.5 mM CaCl_2_, 10 mM HEPES pH 7.4, 8 mM glucose) and maintained in this medium for subsequent experiments. Medium that lacked CaCl_2_ and included 0.4 mM EGTA was used for experiments requiring depletion of extracellular Ca^2+^. To measure Ca^2+^ responses, Cal-520™ fluorescence was recorded using an inverted fluorescence microscope (Nikon Eclipse Ti) coupled to a photometrics CoolSnap MYO camera (180 consecutive images of the cells were recorded, with each image to be taken every second). Mean fluorescence values were extracted for single cells using ImageJ software (1.48v) and plotted against time to generate a kinetic profile. The amplitude of the first peak was calculated by deducting the minimum intensity value from the maximum intensity value and then dividing by the minimum intensity value (see Fig. S7 for examples).

### Coating of magnetic beads

Superparamagnetic beads (4.5 μm diameter; 10^7^; Dynabeads) conjugated to goat anti-mouse-IgG, sheep anti-rat-IgG antibodies (Invitrogen; 200 μg ml^−1^) were coated non-covalently with the antibodies of interest or covalently to poly-D-lysine (Sigma; 200 μg ml^−1^). They were washed with phosphate-buffered saline containing 0.1% (w/v) bovine serum albumin and 0.5 M EDTA (pH 7.4) and resuspended in serum-free M199 media.

### Magnetic tweezers

A mild steel-cored electromagnet was built in-house and set into the stage of an inverted fluorescence microscope (Nikon Eclipse Ti) to form a magnetic tweezers platform, in conjunction with 4.5 µm diameter superparamagnetic beads. The microscope stage was immobilised, ensuring that the forces generated by the magnetic tweezers were identical in every image. Magnetic fields were generated by passing electrical current around copper coils wound around a mild steel core and focused over the sample using pole pieces on each side of the imaging region. Automated control of the field profile and direction was achieved with millisecond precision by powering the field from each pole piece independently, via a computer interface. Facing poles are separated by 36 mm, such that there is an 18 mm gap between the face of each pole and the centre of the imaging area.

The force acting on the superparamagnetic bead (which is transferred to the anchoring receptors) is determined by the magnetic properties of the beads and the spatial profile of the magnetic field (Bryan et al., 2010). To calibrate the magnetic field profile, a Gaussmeter (GM7, Hirst) was used to measure the field at each pole (at the centre of the surface facing the sample) and in the imaging position. More detailed calculations of the field profile were made by fitting these experimental data with a computational model generated with the ANSYS software package (https://www.ansys.com/). Taking into account the 3D nature of the electromagnet, the ANSYS program solved the Biot–Savart equation over a finite element mesh to calculate the field profile around the imaging region. Resultant forces from this field profile were calculated using the finite-element method described in Bryan et al. (2010), such that the total force acting on the bead 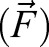 at each position calculated is given by:(1)
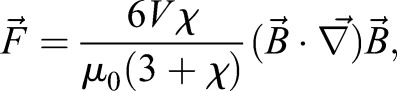
where *V* is the bead volume, *χ* (=3.1) is the magnetic susceptibility of the bead, *μ*_0_ (=4π×10^−7^ NA^−2^) is the permeability of free space, 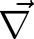 is the mathematical operator nabla, and 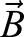 is the magnetic field at the calculated position.

For magnetic tweezer experiments, superparamagnetic beads were precipitated onto confluent HUVECs prepared on 1% fibronectin-coated microdishes (µ-Dish 35 mm ibidi™) at a concentration of 250×10^4^ beads per 50×10^4^ cells per dish. Beads were incubated for 30 min and then unbound beads removed by exchange of medium with HEPES-buffered saline prior to the application of force. Bead movement was recorded using an inverted microscope (Nikon Eclipse Ti) coupled to a photometrics CoolSnap MYO camera and tracked using Spot Tracker plugin of ImageJ.

### Mice

*Itgb1* was deleted from ECs of adult mice (*Itgb1* conditional knockout; *Itgb1*^cKO^). This was carried out by crossing mice containing a tamoxifen-inducible EC-specific Cre (Gothert et al., 2004; Mahmoud et al., 2016) (endothelial-SCL-Cre-ERT) with a strain containing a floxed version of *Itgb1* (*Itgb1^flox/foxl^*). To activate Cre, tamoxifen (Sigma) was administered intraperitoneally for 5 consecutive days (100 mg/kg body weight) (Mahmoud et al., 2016). *Itgb1*^cKO^ mice (female, aged 8–12 weeks) were killed 10 days after the first injection and systematically compared with control littermates treated under the same conditions. All mice were used in accordance with UK legislation [1986 Animals (Scientific Procedures) Act] and experiments were carried out under UK Home Office Project Licence (P28AA2253) for experimentation.

### *En face* staining of murine endothelium

The expression levels of specific proteins were quantified in ECs at regions of the outer curvature (unidirectional flow; disease protected) or inner curvature (bidirectional flow; disease prone) of the murine aortic arch by *en face* staining. Animals were killed by intraperitoneal (i.p.) injection of pentobarbital. Aortae were perfused *in situ* with PBS and then perfusion-fixed with 4% paraformaldehyde prior to staining using 9EG7 and Mab1997 primary antibodies (both at 1:100) and Alexa Fluor 568-conjugated secondary antibodies (Life Technologies) or with Alexa Fluor 568–phalloidin (ThermoFisher Scientific). ECs were co-stained using anti-PECAM-1 antibody (1:100, clone: MEC 13.3, BioLegend), conjugated to FITC fluorophore. DAPI (Sigma) was used to identify nuclei. Stained vessels were analysed using super-resolution confocal microscopy (Zeiss LSM880 AiryScan Confocal). As experimental controls for specific staining, isotype-matched monoclonal antibodies raised against irrelevant antigens were used. The expression of total and active β1 integrin was assessed by quantification of fluorescence intensity for multiple cells using ImageJ software (1.48v). Endothelial cell alignment was quantified by measuring the angle of the major axis of the nucleus in multiple cells (20 per field of view) as previously described (Poduri et al., 2017).

### SMD simulations

SMD simulations were performed with GROMACS 4.6 using the GROMOS96 53a6 force field. The αVβ3 ectodomain structure (PDB 3IJE) was used to run the simulations (Xiong et al., 2009). This structure was chosen because a 3D structure of the ectodomain of a β1-containing integrin is not available. Note that the αV at residues 839–867 region that is missing from the crystal structure of the αVβ3 ectodomain is also missing from our model and that the unstructured regions αV at residues 955–967 and β3 at residues 685–695 were removed for the simulations. The Parrinello–Rahman barostat (Parrinello and Rahman, 1981) was used for pressure coupling with isotropic pressure coupling. The V-rescale thermostat (Bussi et al., 2007) was used for temperature coupling. The Particle Mesh Ewald (PME) algorithm (Darden et al., 1993) was used to model long-range electrostatic interactions and the LINCS algorithm (Hess et al., 1997) was used to constrain bond lengths. The integrin was positioned in the simulation box with the Calf-1 and Calf-2 domains in a tilted (∼30°) orientation relative to the *xy* plane (Fig. 3). This is believed to be the inactive orientation of an integrin. The simulation box size was ∼29.4×∼29.4×∼19.3 nm and the system contained 1586961 atoms. The simulation system was solvated with water molecules, and 150 nM of NaCl was added to neutralise the systems. Subsequently, the system was equilibrated for 2 ns with the protein Cα atoms restrained, followed by SMD simulations. A time step of 2 fs was used for the SMD simulations. The temperature of the simulations was 310 K. The GROMACS pull code was used (pull=constant force) to apply a constant force on the centre of mass of the βA domain. The force was parallel to the *xy* plane of the simulation box. During the SMD simulations the Cα atoms of the αV Calf-2 domain (residues 743–954) were restrained in all directions and the Cα atoms of the β3 β-tail domain (residues 605–684) were restrained in the *z* direction to mimic the cell membrane. Three simulations were run using a force of 200 kJ mol^−1^ nm^−1^ for 80 ns each. Owing to the size of the system at ∼1.5 M particles, these forces, albeit higher than the force integrins may experience in the cell, enabled us to investigate the structural and molecular rearrangements that shear stress causes in the integrin at a reasonable timescale.

### Statistical analysis

Measurements in individual cells were made from 50 cells for each experimental condition in flow studies and from 15–30 cells for each experimental condition in magnetic tweezer studies. Statistics were performed using a paired Student's *t*-test or ANOVA (multiple comparisons, type and post-test is as described in figure legends) in GraphPad Prism 6. Differences between means were considered significant when *P*<0.05. Data are represented as means±s.e.m. *P<0.05, **P<0.01, ***P<0.001.

## Supplementary Material

Supplementary information
