# Supplementary material for: β1 integrin is a sensor of blood flow direction
Source: J Cell Sci. 2019 Jun 3;132(11):jcs229542. doi: 10.1242/jcs.229542 (PMC6589088; doi:10.1242/jcs.229542)
Supplement: Supplementary information [file joces-132-229542-s1.pdf]

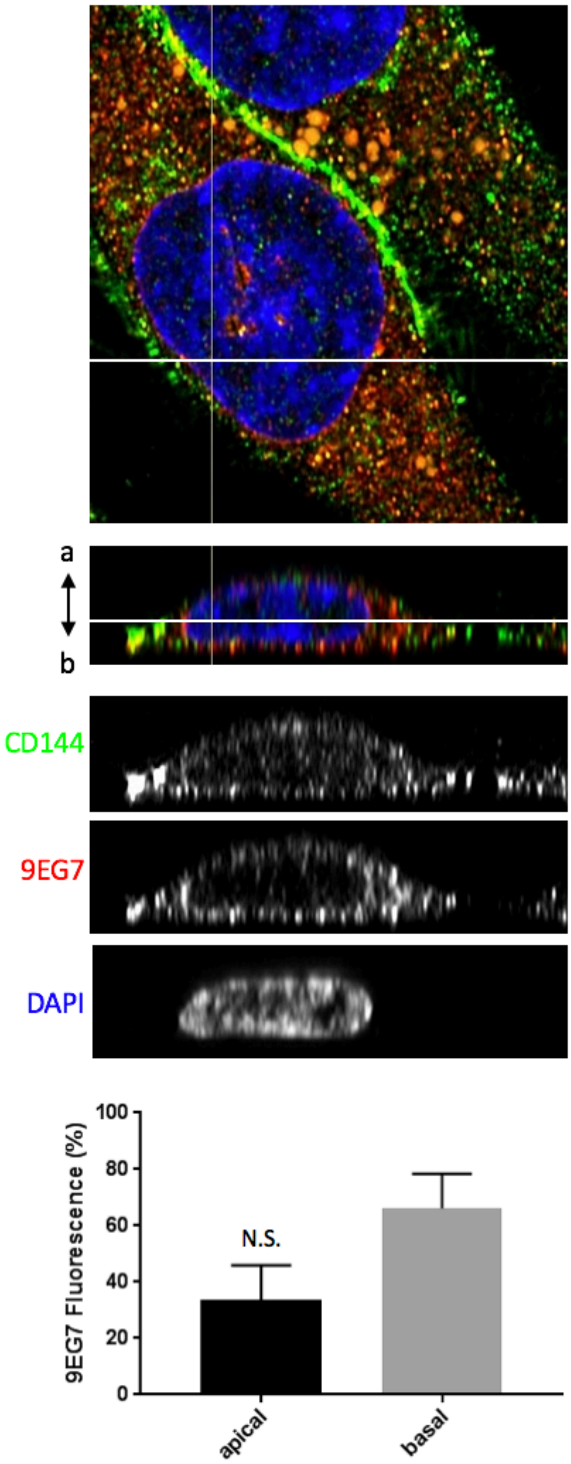

Figure S1. HUVECs were exposed to unidirectional flow for 3 min and then stained with antibodies targeting active β1 integrins (9EG7; red), CD144 (VE-cadherin; green) and DAPI (nuclei; blue). Representative super-resolution confocal microscopy images are shown. 9EG7 mean fluorescence values ± SEM are shown. N.S. = not significant.

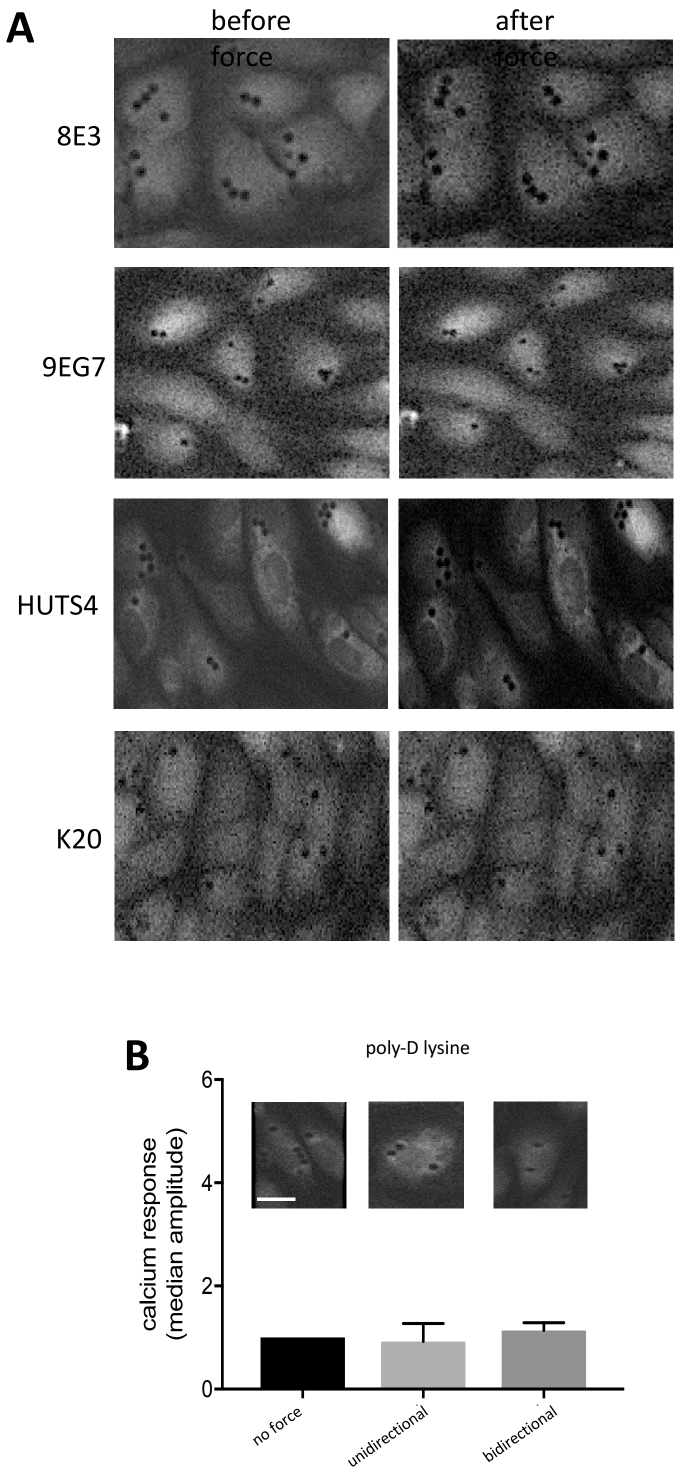

Figure S2. Controls for magnetic tweezers experiments. (A) Beads coated with antibodies targeting β1 integrins remained attached to HUVEC in the presence of magnetic force. HUVECs were loaded with the calcium fluorescent dye Cal-520 and then incubated with beads coated with 8E3, 9EG7, HUTS4 or K20 antibodies. The position of the beads was recorded by fluorescent microscopy before and after the application of unidirectional force (~16 pN) for 2 min. (B) Force application to poly-D lysine coated beads did not activate calcium signalling. HUVECs were loaded with Cal-520 and then incubated with beads coated with poly-D lysine. Beads were exposed to unidirectional force (~16 pN), bidirectional force (1Hz ~16 pN) or no force. Calcium responses were recorded for 3 min using fluorescence microscopy. Representative images are shown. Data were pooled from 5 independent experiments. The median amplitude of the first peak of the calcium response was calculated. Values are shown as means ± SEM.

Journal of Cell Science • Supplementary information

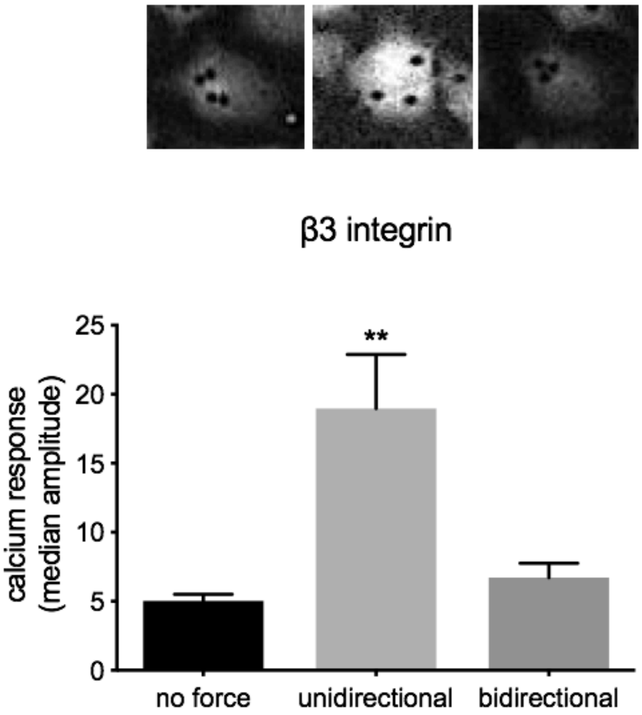

Figure S3. β3 integrins induce Ca^{2+} accumulation in response to unidirectional but not bidirectional shearing force. HUVECs were loaded with Cal-520 and then incubated with beads coated with antibodies targeting β3 integrins (7E3). Beads were exposed to unidirectional force (~16 pN), bidirectional force (1Hz ~16 pN) or no force. Calcium responses were recorded for 3 min using fluorescence microscopy. Representative images are shown. Data were pooled from 5 independent experiments. The median amplitude of the first peak of the calcium response was calculated. Values are shown as means ± SEM and differences were tested using a one-way ANOVA test, with Tukey's test for multiple comparisons. **p<0.01.

Journal of Cell Science • Supplementary information

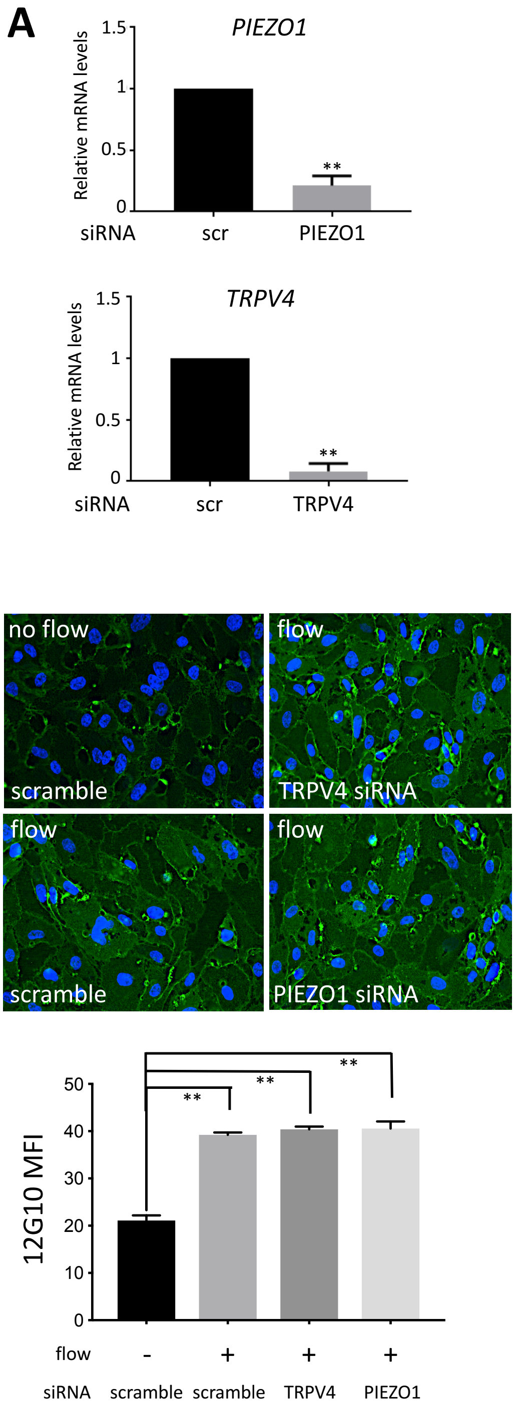

Figure S4. β1 integrin activation by unidirectional flow is not regulated by TRPV4 or Piezo1. (A) qRT-PCR validation of silencing of Piezo1 and TRPV4. HUVECs were transfected with siRNA targeting Piezo1, TRPV4 or with scrambled (scr) sequences as a control. After 72 h, the expression levels of Piezo1 and TRPV4 mRNA were assessed by qRT-PCR. Mean expression levels +/- SEM is shown. Data were pooled from three independent experiments. Differences between means were analysed using a paired t-test. **P<0.01. (B) HUVECs were transfected with siRNA targeting Piezo1, TRPV4 or with scrambled sequences as a control. After 72 h, cells were exposed to unidirectional flow for 3 min or remained under static conditions. Cells were stained with antibodies targeting active β1 integrins (12G10; green) and DAPI (nuclei; blue). Representative images and 12G10 mean fluorescence values ± SEM are shown. Data were pooled from 3 experiments. Differences were analysed using a one-way ANOVA with Tukey's test for multiple comparisons. **p<0.01.

Journal of Cell Science • Supplementary information

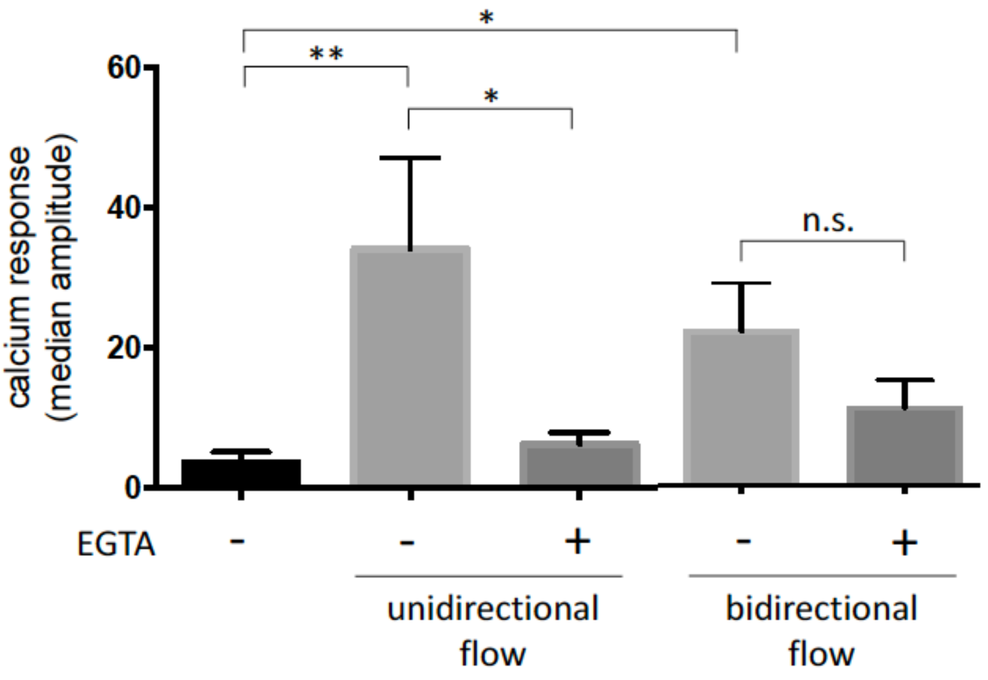

Figure S5. Unidirectional flow induces Ca^{2+} accumulation via external sources. HUVECs were loaded with the calcium fluorescent dye (Cal-520). Some cultures were treated with EGTA to remove calcium from the extracellular media or were left untreated as a control. HUVECs were then exposed to unidirectional or 1 Hz bidirectional flow as indicated and calcium responses were recorded for 3 min using fluorescence microscopy. Data were pooled from 4 independent experiments. The median amplitude of the first peak of the calcium response was calculated. Values are shown as means ± SEM and differences were tested using a two-way ANOVA. *$p<0.05$, **$p<0.01$.

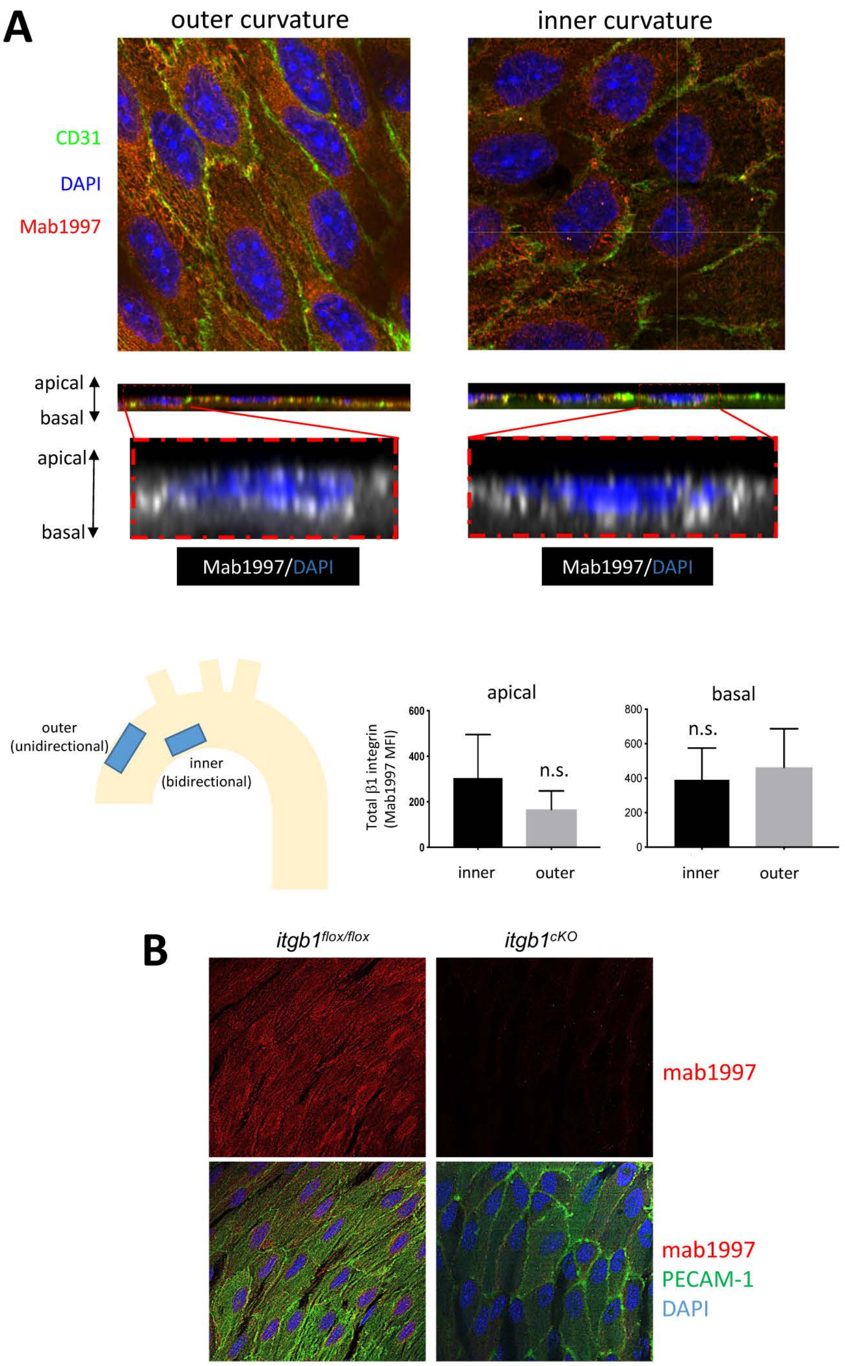

Figure S6. Total β1 integrin staining. Mouse aortic arches were stained *en face* with antibodies targeting β1 integrins (Mab1997; red). EC were co-stained using anti-PECAM-1 antibodies (green) and nuclei were counterstained using DAPI (blue). (A) Wild-type C57BL/6 mice were studied. Fluorescence was measured at the outer curvature (unidirectional flow) and inner curvature (bidirectional flow) regions using super-resolution confocal microscopy. Representative z-series stacks of images are shown indicating apical and basal surfaces. Mean levels at apical and basal regions were calculated at inner and outer curvatures. The majority of the pool of β1 integrin localised to the basal surface but a proportion was also detected at the apical surface at both the inner and outer curvature. (B) *Itgb1*^{cKO} mice were generated by crossing *itgb1*$^{fl/fl}$ with a transgenic strain expressing a tamoxifen-activated version of Cre in EC (endothelial-SCL-Cre-ERT) prior to administration of tamoxifen for 5 days. To validate genetic deletion, β1 integrin expression was assessed in the descending aorta by *en face* staining using Mab1997 antibodies (red). EC were co-stained using anti-PECAM-1 antibodies (green) and nuclei were counterstained using DAPI (nuclei; blue). Representative images are shown.

Journal of Cell Science • Supplementary information

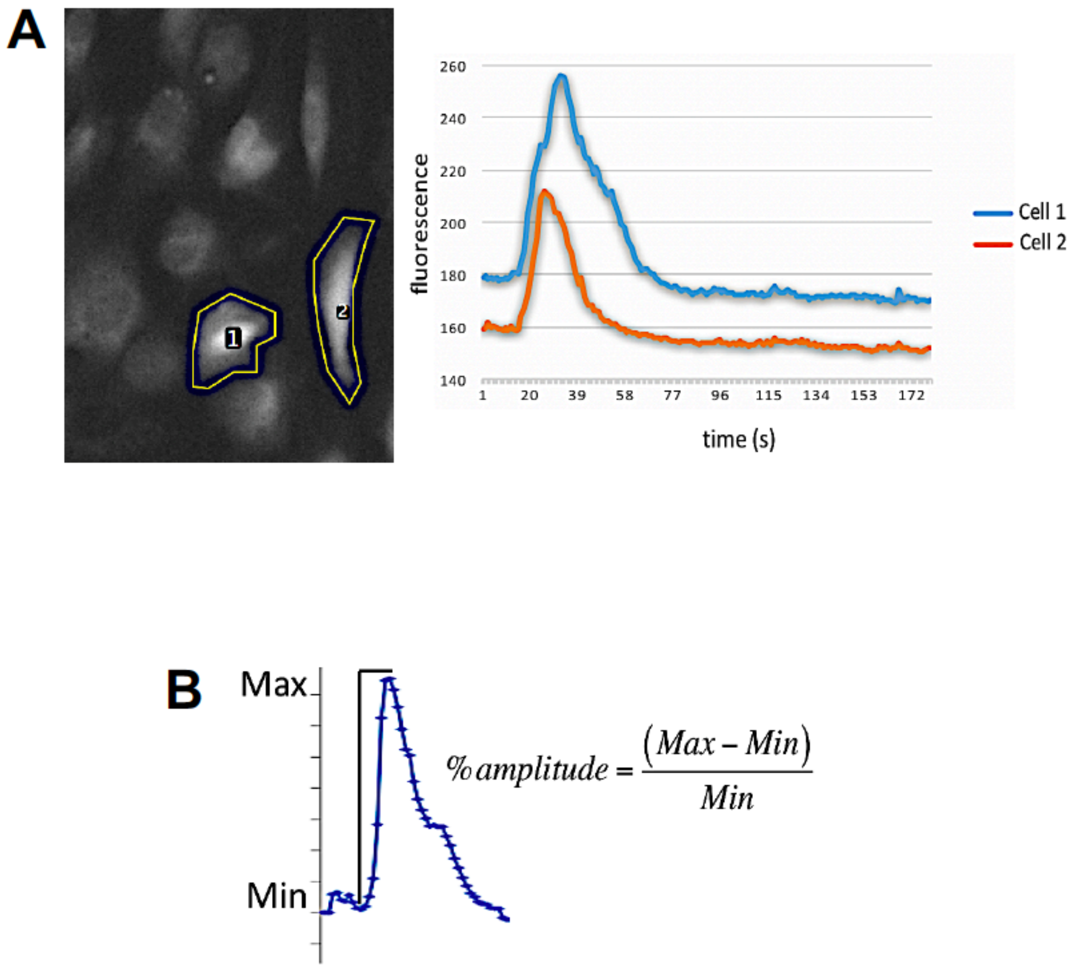

Figure S7. Examples of calcium response quantitation in single cells. (A) HUVECs were loaded with Cal-520 and exposed to unidirectional flow. Calcium responses were recorded for 3 min using fluorescence microscopy. Using ImageJ software, the mean fluorescent intensity (y axis) for individual cells was measured each second (x axis). Representative images (left panel) and fluorescence data from two cells (right panel) are shown. (B) The peak amplitude for each cell was calculated by deducting the minimum intensity value from the maximum intensity value and then dividing by the minimum intensity value (peak amplitude= (max-min)/min).

Journal of Cell Science • Supplementary information

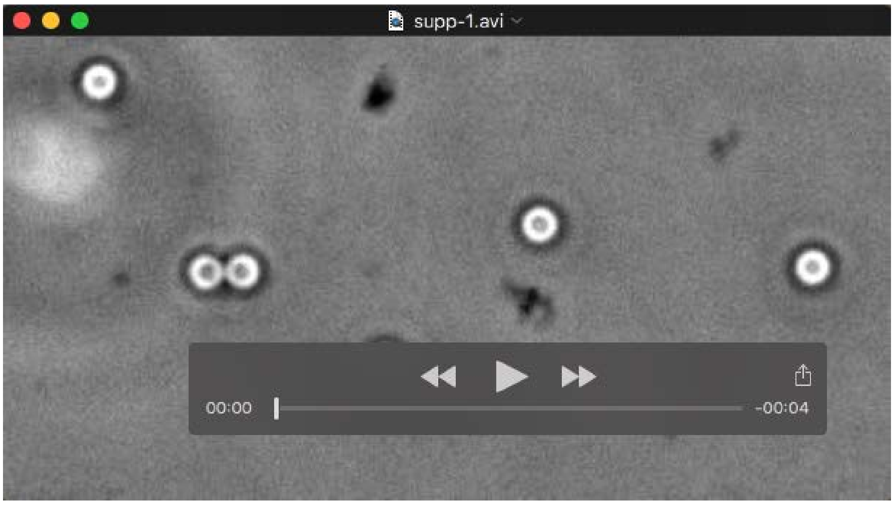

Movie 1. Magnetic tweezers displaced paramagnetic beads in suspension. The ability of the magnetic tweezers system to generate magnetic force was tested by monitoring displacement of suspended superparamagnetic beads. Beads were recorded prior and after the application of unidirectional force (at 2 s).

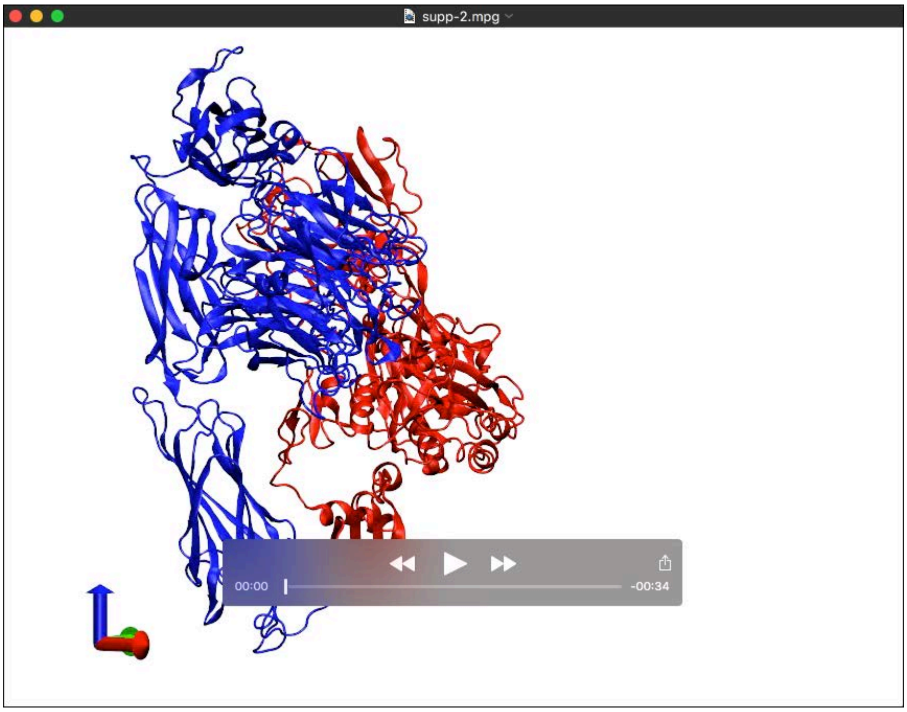

Movie 2. Molecular dynamic simulations of the αVβ3 integrin exposed to unidirectional force. The ectodomain of the αVβ3 integrin (αV is shown in blue and β3 integrin is shown in red) in which a 200 kJ mol^{-1} nm^{-1} force parallel to the membrane was applied on the βA domain of the head region of the inactive form. Water and ions are omitted for clarity. The integrin Calf-2 and β-tail domains were restrained to mimic the cell membrane (see Methods for more details).

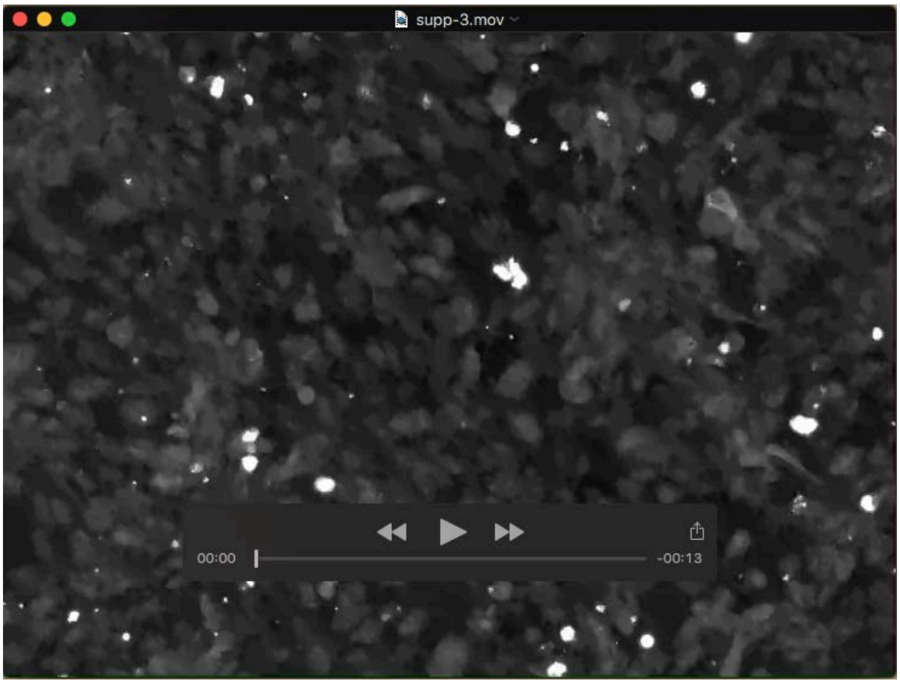

Movie 3. Example of calcium response to unidirectional flow. HUVECs were loaded with Cal-520 and exposed to unidirectional flow for 3 min. Calcium responses were recorded for 3 min using fluorescence microscopy. Data shown are representative of 15 independent experiments that gave closely similar results.

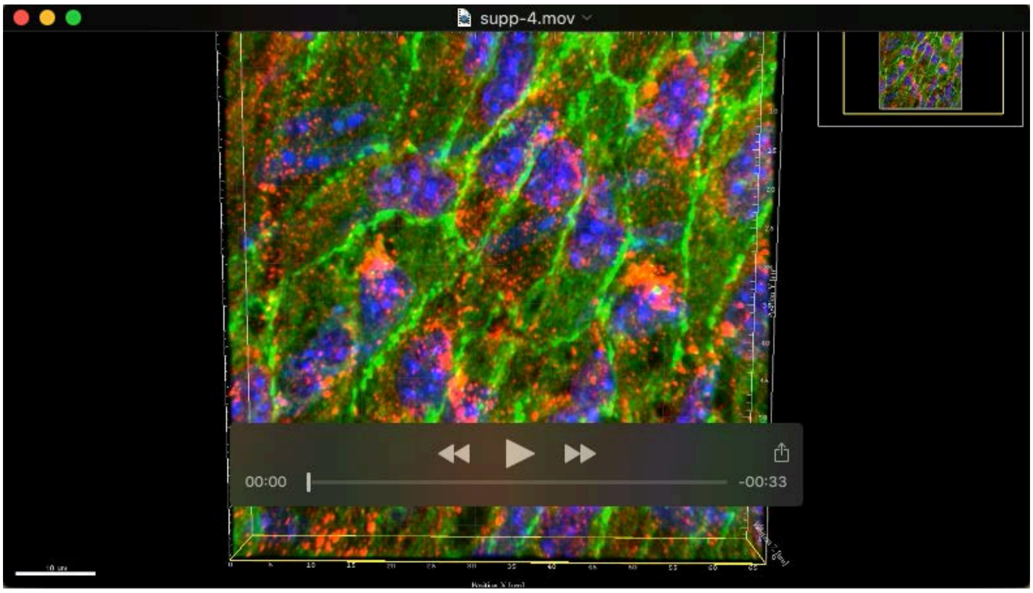

Movie 4. EC at the outer curvature of the aortic arch express active β1 integrins at the apical surface. Mouse aortic arches were stained *en face* with antibodies targeting active β1 integrins (9EG7; red). EC were co-stained using anti-PECAM-1 antibodies (green) and nuclei were counterstained using DAPI (nuclei; blue). Fluorescence was measured at the outer curvature region using super-resolution confocal microscopy. A z-series stack of images was generated and represented in 3D using Imaris software. Note the presence of red signal (active β1 integrins) on the apical (upper) and basal (lower) surfaces.
